# The International Congress Will Be Held in the United States

**Published:** 1885-10-20

**Authors:** 


THE INTERNATIONAL CONGRESS WILL BE HELD IN THE UNITED STATES. —The Journal of the American Medical Association states that the action of the committee of arrangements, as finally adopted at New York, and the organization for which they provide, are free from every objection that has been urged against the previous work of the committee, and that the Ninth International Medical Congress will be held in the city of Washington, in 1887, and will be equal, in all respects, to any previous meeting of the Congress.

